# L-DOPA enhances neural direction signals in younger and older adults

**DOI:** 10.1016/j.neuroimage.2022.119670

**Published:** 2022-12-01

**Authors:** Christoph Koch, Christian Baeuchl, Franka Glöckner, Philipp Riedel, Johannes Petzold, Michael N. Smolka, Shu-Chen Li, Nicolas W. Schuck

**Affiliations:** a Max Planck Research Group NeuroCode, Max Planck Institute for Human Development, Berlin, Germany; b International Max Planck Research School on the Life Course, Max Planck Institute for Human Development, Berlin, Germany; c Faculty of Psychology, Chair of Lifespan Developmental Neuroscience, Technische Universität Dresden, Dresden, Germany; d Department of Psychiatry, Technische Universität Dresden, Dresden, Germany; e Centre for Tactile Internet with Human-in-the-Loop (CeTI), Technische Universität, Dresden, Germany; f Max Planck UCL Centre for Computational Psychiatry and Aging Research, Berlin, Germany; g Institute of Psychology, Universität Hamburg, Hamburg, Germany

**Keywords:** Spatial navigation, Aging, Neural dedifferentiation, Tuning functions, fMRI, MVPA, Dopamine

## Abstract

Previous studies indicate a role of dopamine in spatial navigation. Although neural representations of direction are an important aspect of spatial cognition, it is not well understood whether dopamine directly affects these representations, or only impacts other aspects of spatial brain function. Moreover, both dopamine and spatial cognition decline sharply during age, raising the question which effect dopamine has on directional signals in the brain of older adults. To investigate these questions, we used a double-blind cross-over L-DOPA/Placebo intervention design in which 43 younger and 37 older adults navigated in a virtual spatial environment while undergoing functional magnetic resonance imaging (fMRI). We studied the effect of L-DOPA, a dopamine precursor, on fMRI activation patterns that encode spatial walking directions that have previously been shown to lose specificity with age. This was done in predefined regions of interest, including the early visual cortex, retrosplenial cortex, and hippocampus. Classification of brain activation patterns associated with different walking directions was improved across all regions following L-DOPA administration, suggesting that dopamine broadly enhances neural representations of direction. No evidence for differences between regions was found. In the hippocampus these results were found in both age groups, while in the retrosplenial cortex they were only observed in younger adults. Taken together, our study provides evidence for a link between dopamine and the specificity of neural responses during spatial navigation.

**Significance Statement:**

The sense of direction is an important aspect of spatial navigation, and neural representations of direction can be found throughout a large network of space-related brain regions. But what influences how well these representations track someone’s true direction? Using a double-blind cross-over L-DOPA/Placebo intervention design, we find causal evidence that the neurotransmitter dopamine impacts the fidelity of direction selective neural representations in the human hippocampus and retrosplenial cortex. Interestingly, the effect of L-DOPA was either equally present or even smaller in older adults, despite the well-known age related decline of dopamine. These results provide novel insights into how dopamine shapes the neural representations that underlie spatial navigation.

## 1 Introduction

A role of dopamine (DA) in spatial navigation is well established. Anatomically, spatial cognition depends on a network of brain regions centered around the hippocampus (HC) and retrosplenial cortex (RSC) (Burgess, Maguire, O’Keefe, 2002, Chersi, Burgess, 2015), both of which are targets of dopaminergic innervation (Berger, Verney, Alvarez, Vigny, Helle, 1985, McNamara, Dupret, 2017). Behaviorally, spatial navigation abilities are influenced by DA functioning in younger as well as older animals and humans (El-Ghundi, Fletcher, Drago, Sibley, O’Dowd, George, 1999, Granado, Ortiz, Suárez, Martín, Ceña, Solís, Moratalla, 2008, Kentros, Agnihotri, Streater, Hawkins, Kandel, 2004, Thurm, Schuck, Fauser, Doeller, Stankevich, Evens, Riedel, Storch, Lueken, Li, 2016).

Much less is known about how DA might change the neural representations that support spatial navigation. Particularly interesting for human neuroscience are direction selective representations (Taube, 2007), which have been found, amongst others, in the HC, the RSC and visual cortex (Cacucci, Lever, Wills, Burgess, O’Keefe, 2004, Flossmann, Rochefort, 2021, Guitchounts, Masís, Wolff, Cox, 2020, Shine, Valdés-Herrera, Hegarty, Wolbers, 2016), and can be decoded from human fMRI signals (Koch et al., 2020). We hypothesized that DA affects direction encoding in the human brain and tested this idea using a double-blind placebo controlled intervention design. Specifically, we predicted that oral administration of L-DOPA, a dopamine precursor, would influence how accurately walking direction can be decoded from multi-voxel fMRI patterns in the above named ROIs.

Next to its role in spatial navigation, DA has also received much attention in the context of aging, where reduced DA functions are prevalent and are thought to underlie age-related cognitive declines (Bäckman, Nyberg, Lindenberger, Li, Farde, 2006, Chowdhury, Guitart-Masip, Lambert, Dayan, Huys, Düzel, Dolan, 2013, Li, Lindenberger, Bäckman, 2010, Volkow, Gur, Wang, Fowler, Moberg, Ding, Hitzemann, Smith, Logan, 1998). Computational models have shown that declining neuromodulatory effects of DA lead to losses in the signal-to-noise ratio of neural responses (Cohen, Servan-Schreiber, 1992, Servan-Schreiber, Printz, Cohen, 1990), which in the aging brain can lead to neural representations that are less specific or “dedifferentiated” (Li, Lindenberger, Sikström, 2001, Li, Rieckmann, 2014). In line with these models, dedifferentiation has repeatedly been observed in older adults (OA) at the behavioral and neural levels (Carp, Park, Hebrank, Park, Polk, 2011, Carp, Park, Polk, Park, 2011, Koch, Li, Polk, Schuck, 2020, Li, Lindenberger, Hommel, Aschersleben, Prinz, Baltes, 2004, Park, Polk, Park, Minear, Savage, Smith, 2004). Neural dedifferentiation, in turn, has been linked to decreased memory performance (Koen, Hauck, Rugg, 2019, Sommer, Fandakova, Grandy, Shing, Werkle-Bergner, Sander, 2019, St-Laurent, Abdi, Bondad, Buchsbaum, 2014), establishing an explanatory link between DA, neural representations and cognitive aging.

These roles of DA in spatial navigation and aging might contribute to the pronounced decline in spatial cognition with age (Lester, Moffat, Wiener, Barnes, Wolbers, 2017, Moffat, 2009, Schuck, Doeller, Polk, Lindenberger, Li, 2015, Wolbers, Dudchenko, Wood, 2014), and to the neural dedifferentiation of direction-selective (Koch et al., 2020) and hippocampal signals (Schuck et al., 2015) in the aging brain. Moreover, since the sharp decline of DA with age should lead to lower baseline availability of DA in OA, the effects of DA might be stronger in OA relative to younger adults (YA) – reflecting DA’s inverted-U-shape relation to cognitive performance (Cools, D’Esposito, 2011, Li, Lindenberger, Bäckman, 2010, Li, Papenberg, Nagel, Preuschhof, Schröder, Nietfeld, Bertram, Heekeren, Lindenberger, Bäckman, 2013, Vijayraghavan, Wang, Birnbaum, Williams, Arnsten, 2007). Indeed, one previous study found age-related effects of the DA receptor agonist bromocriptine on dedifferentiation in the HC (Abdulrahman et al., 2017). Moreover, HC-dependent episodic memory, spatial navigation, and learning have been found to be affected by genetic polymorphisms related to dopamine D2 receptor availability (COMT Val158Met, C957T CC; Li, Papenberg, Nagel, Preuschhof, Schröder, Nietfeld, Bertram, Heekeren, Lindenberger, Bäckman, 2013, Papenberg, Bäckman, Nagel, Nietfeld, Schröder, Bertram, Heekeren, Lindenberger, Li, 2014) or hippocampal function (KIBRA SNP rs17070145; Schuck, Doeller, Schjeide, Schröder, Frensch, Bertram, Li, 2013, Schuck, Petok, Meeter, Schjeide, Schröder, Bertram, Gluck, Li, 2018) in OA, but not YA. Based on these findings, we therefore also tested whether L-DOPA effects on walking direction decoding would be stronger in OA relative to YA.

Finally, we expected that DA could also influence the shape of population-based tuning functions of direction. Although direction-sensitive cells often have a preferred direction, they also fire in response to non-preferred directions in proportion to their similarity to the preferred direction (Taube, 2007). Hence, encoding of direction information seems to follow a Gaussian tuning function, in particular on a population level (Averbeck et al., 2006). Research has also shown that age-related neural dedifferentiation results in increased width of such tuning functions with age (Leventhal, Wang, Pu, Zhou, Ma, 2003, Liang, Yang, Li, Zhang, Wang, Zhou, Leventhal, 2010, Schmolesky, Wang, Pu, Leventhal, 2000), which we too have reported previously using fMRI (Koch et al., 2020). We therefore also investigated whether L-DOPA has effects on the precision of fMRI-derived tuning functions of direction information and whether such effects may interact with age.

## 2 Materials and methods

### 2.1 Participants

This study was part of a larger project in which the same participants performed multiple tasks, including a sequential decision making task and a virtual reality spatial memory task inside the scanner and other decision tasks outside of the scanner.

Here, we only report results from the MRI analysis of the VR task described below. Specifically, following our previous publication (Koch et al., 2020), our analyses were specific to neural representations of direction signals during the spatial memory task performed while undergoing fMRI. Other data from the same participants was not within the purview of this study and was therefore not investigated. Data of 102 participants which were recruited for two MRI sessions and randomly assigned to one of the two drug intervention groups (i.e., L-DOPA – Placebo or Placebo – L-DOPA) was available for investigating our research question. Eighty-eight of these participants (43 OA, 45 YA) successfully completed both sessions without technical errors. Four additional OA were excluded from further analyses because they did not respond in at least a third of the trials in at least one of the two sessions. Decoding analyses of the L-DOPA effects introduced additional requirements for the distribution of walking direction (see Materials and Methods) that were not met for four participants (2 OA, 2 YA). Thus, the final effective sample for these analyses also excluded these participants and comprise of a total of 37 OA (age 65–75, 6 female) and 43 YA (age 26–35, 16 female).

Note that the relatively low number of female OA reflects difficulties in recruitment after the onset of the COVID-19 pandemic.

### 2.2 Virtual reality task

During each session of fMRI data collection participants had to complete a similar variant of a spatial memory task that was used in previous studies (Schuck, Doeller, Polk, Lindenberger, Li, 2015, Thurm, Schuck, Fauser, Doeller, Stankevich, Evens, Riedel, Storch, Lueken, Li, 2016). Analyses of the present work are mainly concerned with directional signals obtained during free navigation, and hence focus on the corresponding task phases. Specifically, to avoid effects of changed environmental cues on directional signals (e.g. Taube et al., 1990) or initial learning, we considered only data from the feedback phase for this study (see below). On average, the included data reflected a period of 17.36 min from free navigation per session.

**Fig. 1:**
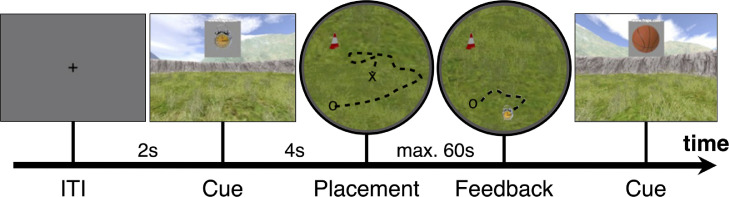
Task procedure during feedback phase. Each trial started with a fixation cross on a grey background for two seconds. Afterwards a cue was presented showing the object to which participants needed to navigate (object locations were learned during encoding phase). The participant then had 60 s to navigate from their starting location (cross) to the object location according to their spatial memory. Participants indicated that they had arrived at the remembered location (circle) by pressing a response button, after which the object appeared at its true location. Participants could observe the difference between their response and the correct location and were required to navigate towards and walk over the correct location, before the cue of the next trial was presented.

Briefly, participants were placed in a virtual, circular arena in which they could move around freely using a custom-made MRI-compatible joystick. The arena consisted of a circular grass plane surrounded by a wall. Participants could also see distal cues (mountains, clouds) as well as a local cue (traffic cone) to aid orientation (see Fig. 1). We asked participants to remember the location of five objects within the 360${}^{\circ }$ arena. First, an initial encoding phase took place in which participants could see and walk to the locations of all objects appearing one after the other. Learning of object location then continued in a feedback phase: participants were placed close to the center of the arena with a random heading direction. After the brief presentation of a grey screen and fixation cross, a picture of the first object was shown. Participants were asked to navigate as closely as possible to the location of this object and indicate their final position with a button press within a maximum of 60 s. To provide feedback, the true object location was shown to participants following their response, and they were then asked to navigate to and walk over the shown location. After the feedback, participants were shown another object and the procedure repeated without placing the player in the center of the arena until all five objects were completed. The order in which the five objects were shown was pseudo-randomized. Once all five objects were completed, participants were again placed close to the arena’s center and had to navigate to all five objects in the same manner for a total of six repetitions (i.e., 5 × 6 = 30 feedback trials). In a final transfer phase of the task (data not analyzed in this study, see above), either the arena size or the location of the traffic cone were altered, and participants‘ object location memory was tested again as above. For the second session participants had to learn the location of five different objects, but the trial structure and procedures were identical otherwise. Completing one session took participants between 14 and 49 min.

### 2.3 Drug administration

Following a double-blind drug administration design, participants were given either a total of 225 mg of L-DOPA (Madopar, Roche, Levodopa/Benserazid, 4:1 ratio) or a placebo (P-Tabletten white 8 mm Lichtenstein, Winthrop Arzneimittel) before each MRI session in the form of two orally administered dosages. A first dosage (150 mg L-DOPA/Placebo) was given about 10 min before subjects entered the MRI scanner, roughly one hour before the spatial navigation task began. To assure high dopamine availability during the task, a second booster dosage (75 mg L-DOPA/Placebo) was administered roughly ten minutes before task onset (cf. Kroemer et al., 2019). Participants were pseudo-randomly assigned to one of two groups with different session order, either the group that received L-DOPA in the first session and placebo in the second session (Drug-Placebo group, 40 subjects) or the group that started with the placebo in the first session (Placebo-Drug group, 44 participants).

### 2.4 Image acquisition

All data was collected on a 3 Tesla Siemens Magnetom Trio (Siemens, Erlangen, Germany) MRI scanner. T1-weighted structural images were collected at the beginning of the first session using a MP-RAGE pulse sequence (0.8×0.8×0.8mmvoxels, TR=2400ms, TE=2.19ms, TI=1000ms, acquisitionmatrix=320×320×240, FOV=272mm, $\text{flip}\;\text{angle}=8^{\circ }$, $\text{bandwidth}=210\;\frac{\text{Hz}}{\text{Px}}$). At the beginning of the second session T2-weighted structural scan was collected (0.8×0.8×0.8mmvoxels, TR=3200ms, TE=565ms, acquisitionmatrix=350×350×2630, FOV=272mm, $\text{bandwidth}=744\;\frac{\text{Hz}}{\text{Px}}$).

Functional on-task data was collected using a T2*-weighted echo-planar imaging (EPI) pulse sequence 3×3×2.5mmvoxels, slicethickness=2.5mm, distancefactor=20%, TR=2360ms, TE=25ms, imagematrix=64×64, FOV=192mm, $\text{flip}\;\text{angle}=80^{\circ }$, 48axialslices, GRAPPAparallelimaging, accelerationfactor:2, interleavedacquisition). The sequence lasted until the task was completed and took about 15–50 min. Additional functional scans not analyzed in this manuscript included data from the transfer phase, data from a decision making task, as well as data from a resting state scan collected at the start of each session.

Quality of all collected functional sequences was assessed using MRI quality control (MRIQC; Esteban et al., 2017). The quality measure of framewise displacement (FD, threshold 3 mm), a measure for movement during image acquisition (Power et al., 2014), was extracted and used for statistical control.

### 2.5 ROIs

Each ROI was created from anatomical labels obtained from Mindboggle’s FreeSurfer-based segmentation of each participant’s individual T1-weighted images (Klein et al., 2017). We investigated three predefined ROIs in light of previous findings indicating direction selective coding in these regions (Cacucci, Lever, Wills, Burgess, O’Keefe, 2004, Flossmann, Rochefort, 2021, Guitchounts, Masís, Wolff, Cox, 2020, Koch, Li, Polk, Schuck, 2020, Shine, Valdés-Herrera, Hegarty, Wolbers, 2016, Taube, 2007). An early visual cortex (EVC) ROI, consisting of the bilateral cortical masks of the cuneus, lateral occipital cortex, and the pericalcarine cortex (mean number of voxels: 1480.87). A ROI of the retrosplenial cortex (RSC) constructed from the bilateral, cortical masks of the cingulate ishtmus (mean number of voxels: 198.55). A mask of the hippocampus (HC) was extracted from the respective bilateral masks of the parcellation (mean number of voxels: 323.64). In addition to these core masks, we added a ROI of the left motor cortex, constructed from the cortical mask of the left precentral gyrus, to serve as a control (mean number of voxels: 555.45). Although our resolution was suboptimal to investigate small areas, we included a mask of the entorhinal cortex (EC, mean number of voxels: 174.09) in order to explore if direction signals could be found there as well (see Inline Supplementary Table S1 for all ROI sizes).

### 2.6 Image preprocessing

*Copyright Waiver*

Results included in this manuscript come from preprocessing performed using *fMRIPrep* 20.0.6 (Esteban, Blair, Markiewicz, Berleant, Moodie, Ma, Isik, Erramuzpe, Kent, Goncalves, DuPre, Sitek, Gomez, Lurie, Ye, Poldrack, Gorgolewski, 2018, Esteban, Markiewicz, Blair, Moodie, Isik, Erramuzpe Aliaga, Kent, Goncalves, DuPre, Snyder, Oya, Ghosh, Wright, Durnez, Poldrack, Gorgolewski, 2018; RRID:SCR_016216), which is based on *Nipype* 1.4.2 (Gorgolewski, Burns, Madison, Clark, Halchenko, Waskom, Ghosh, 2011, Gorgolewski, Esteban, Markiewicz, Ziegler, Ellis, Notter, Jarecka, Johnson, Burns, Manhães-Savio, Hamalainen, Yvernault, Salo, Jordan, Goncalves, Waskom, Clark, Wong, Loney, Modat, Dewey, Madison, Visconti di Oleggio Castello, Clark, Dayan, Clark, Keshavan, Pinsard, Gramfort, Berleant, Nielson, Bougacha, Varoquaux, Cipollini, Markello, Rokem, Moloney, Halchenko, Wassermann, Hanke, Horea, Kaczmarzyk, de Hollander, DuPre, Gillman, Mordom, Buchanan, Tungaraza, Pauli, Iqbal, Sikka, Mancini, Schwartz, Malone, Dubois, Frohlich, Welch, Forbes, Kent, Watanabe, Cumba, Huntenburg, Kastman, Nichols, Eshaghi, Ginsburg, Schaefer, Acland, Giavasis, Kleesiek, Erickson, Küttner, Haselgrove, Correa, Ghayoor, Liem, Millman, Haehn, Lai, Zhou, Blair, Glatard, Renfro, Liu, Kahn, Pérez-García, Triplett, Lampe, Stadler, Kong, Hallquist, Chetverikov, Salvatore, Park, Poldrack, Craddock, Inati, Hinds, Cooper, Perkins, Marina, Mattfeld, Noel, Snoek, Matsubara, Cheung, Rothmei, Urchs, Durnez, Mertz, Geisler, Floren, Gerhard, Sharp, Molina-Romero, Weinstein, Broderick, Saase, Andberg, Harms, Schlamp, Arias, Papadopoulos Orfanos, Tarbert, Tambini, De La Vega, Nickson, Brett, Falkiewicz, Podranski, Linkersdörfer, Flandin, Ort, Shachnev, McNamee, Davison, Varada, Schwabacher, Pellman, Perez-Guevara, Khanuja, Pannetier, McDermottroe, Ghosh, 2018; RRID:SCR_002502). The boilerplate text in this section (2.6) was automatically generated by fMRIPrep with the express intention that users should copy and paste this text into their manuscripts *unchanged*. It is released under the CC0 license.

*Anatomical data preprocessing*

The T1-weighted (T1w) image was corrected for intensity non-uniformity (INU) with N4BiasFieldCorrection (Tustison et al., 2010), distributed with ANTs 2.2.0 (Avants et al., 2008; RRID:SCR_004757), and used as T1w-reference throughout the workflow. The T1w-reference was then skull-stripped with a *Nipype* implementation of the antsBrainExtraction.sh workflow (from ANTs), using OASIS30ANTs as target template. Brain tissue segmentation of cerebrospinal fluid (CSF), white-matter (WM) and gray-matter (GM) was performed on the brain-extracted T1w using fast (FSL 5.0.9; RRID:SCR_002823; Zhang et al., 2001). Brain surfaces were reconstructed using recon-all (FreeSurfer 6.0.1; RRID:SCR_001847; Dale et al., 1999), and the brain mask estimated previously was refined with a custom variation of the method to reconcile ANTs-derived and FreeSurfer-derived segmentations of the cortical gray-matter of Mindboggle (RRID:SCR_002438; Klein et al., 2017). Volume-based spatial normalization to two standard spaces (MNI152Lin, MNI152NLin2009cAsym) was performed through nonlinear registration with antsRegistration (ANTs 2.2.0), using brain-extracted versions of both T1w reference and the T1w template. The following templates were selected for spatial normalization: *Linear ICBM Average Brain (ICBM152) Stereotaxic Registration Model* (Mazziotta et al., 1995; TemplateFlow ID: MNI152Lin), *ICBM 152 Nonlinear Asymmetrical template version 2009c* (Fonov et al., 2009; RRID:SCR_008796; TemplateFlow ID: MNI152NLin2009cAsym).

*Functional data preprocessing*

For each of the 4 BOLD runs collected per subject (two task related runs reported here and 2 resting state runs not reported here), the following preprocessing was performed. First, a reference volume and its skull-stripped version were generated using a custom methodology of *fMRIPrep*. Susceptibility distortion correction (SDC) was omitted. The BOLD reference was then co-registered to the T1w reference using bbregister (FreeSurfer) which implements boundary-based registration (Greve and Fischl, 2009). Co-registration was configured with six degrees of freedom. Head-motion parameters with respect to the BOLD reference (transformation matrices, and six corresponding rotation and translation parameters) are estimated before any spatiotemporal filtering using mcflirt (FSL 5.0.9; Jenkinson et al., 2002). BOLD runs were slice-time corrected using 3dTshift from AFNI 20160207 (Cox and Hyde, 1997; RRID:SCR_005927). The BOLD time-series were resampled onto the following surfaces (FreeSurfer reconstruction nomenclature): *fsnative, fsaverage*. The BOLD time-series (including slice-timing correction when applied) were resampled onto their original, native space by applying the transforms to correct for head-motion. These resampled BOLD time-series will be referred to as *preprocessed BOLD in original space*, or just *preprocessed BOLD*. The BOLD time-series were resampled into standard space, generating a *preprocessed BOLD run in MNI152Lin space*. The first step in this process was that a reference volume and its skull-stripped version were generated using a custom methodology of *fMRIPrep*. Several confounding time-series were calculated based on the *preprocessed BOLD*: framewise displacement (FD), DVARS and three region-wise global signals. FD and DVARS are calculated for each functional run, both using their implementations in *Nipype* (following the definitions by Power et al., 2014). The three global signals are extracted within the CSF, the WM, and the whole-brain masks. Additionally, a set of physiological regressors were extracted to allow for component-based noise correction (*CompCor*; Behzadi et al., 2007). Principal components are estimated after high-pass filtering the *preprocessed BOLD* time-series (using a discrete cosine filter with 128s cut-off) for the two *CompCor* variants: temporal (tCompCor) and anatomical (aCompCor). tCompCor components are then calculated from the top 5% variable voxels within a mask covering the subcortical regions. This subcortical mask is obtained by heavily eroding the brain mask, which ensures it does not include cortical GM regions. For aCompCor, components are calculated within the intersection of the aforementioned mask and the union of CSF and WM masks calculated in T1w space, after their projection to the native space of each functional run (using the inverse BOLD-to-T1w transformation). Components are also calculated separately within the WM and CSF masks. For each CompCor decomposition, the *k* components with the largest singular values are retained, such that the retained components’ time series are sufficient to explain 50 percent of variance across the nuisance mask (CSF, WM, combined, or temporal). The remaining components are dropped from consideration. The head-motion estimates calculated in the correction step were also placed within the corresponding confounds file. The confound time series derived from head motion estimates and global signals were expanded with the inclusion of temporal derivatives and quadratic terms for each (Satterthwaite et al., 2013). Frames that exceeded a threshold of 0.5 mm FD or 1.5 standardised DVARS were annotated as motion outliers. All resamplings can be performed with *a single interpolation step* by composing all the pertinent transformations (i.e. head-motion transform matrices, susceptibility distortion correction when available, and co-registrations to anatomical and output spaces). Gridded (volumetric) resamplings were performed using antsApplyTransforms (ANTs), configured with Lanczos interpolation to minimize the smoothing effects of other kernels Lanczos (1964). Non-gridded (surface) resamplings were performed using mri_vol2surf (FreeSurfer).

Many internal operations of *fMRIPrep* use *Nilearn* 0.6.2 (RRID:SCR_001362; Abraham et al., 2014), mostly within the functional processing workflow. For more details of the pipeline, see the section corresponding to workflows in fMRIPrep’s documentation.

### 2.7 fMRI analyses

*Classification of walking direction*

All classification of walking direction was performed in Python (Python Software Foundation; Python Language Reference, version 3.7.8; available at http://www.python.org) and relied on scikit-learn (Pedregosa et al., 2011) and nilearn (Abraham et al., 2014). Statistical analyses and plotting was performed in R (version 4.0.3, R Core Team, 2021), using the packages lme4 (Bates et al., 2015), emmeans (Lenth, 2021) and ggplot2 (Wickham, 2016). All conducted post-hoc tests, if not specified otherwise, were corrected for multiple comparisons using Tukey correction.

Functional data was prepared for classification by smoothing images with a 3 mm FWHM kernel. Next, nilearn’s signal.clean function was used to detrend, high-pass filter ($\frac{1}{128}\text{Hz}$), de-noise (using 10 components of aCompCor) and z-standardize the time courses.

Participants’ walking direction was extracted from navigated paths within the virtual environment. The complete 360${}^{\circ }$-space of direction was binned into six equally spaced bins of 60${}^{\circ }$. Classifier training examples were then constructed by taking fMRI multi-voxel patterns in response to consistent walking within one binned direction for at least one second. Hence the number of classifier examples for each participant and direction were dependent on the travelled paths and the number of direction changes (for more detail on the number of classifier examples, see SI Section 3). If the same example spanned multiple TRs (i.e., was longer than 2.36s) all TRs spanned were averaged to assure a single voxel-pattern per example. Voxel responses were taken two TRs (4.72s) after the event to adjust for hemodynamic lag. A multinomial logistic regression classifier (L2 regularization, C = 1, tolerance = 10^-4^, 1000 maximum iterations; as implemented in scikit-learn) was applied to the resulting activation patterns in order to test whether walking direction could be classified. Cross-validation was done separately for L-DOPA and placebo sessions.

Each session was split into into three folds, and cross-validated decoding was performed across these folds from the same session. We ensured a balanced number of training examples for each class by upsampling underrepresented classes if necessary. A balanced accuracy score was calculated for each test set and results were pooled across all cross-validation runs. To asses above-chance classification accuracy the resulting scores were tested to exceed a chance baseline (16.66%) using one-sided, one-sample t-tests and one-sided comparisons to permutation distributions. Said distributions resulted from repeating individual classification procedures 1000 times with randomly permuted class labels in the training set. A permutation distribution of sample means was obtained by following the same averaging procedure as for the true values, just for each iteration of the permutation. To test whether classification accuracy was influenced by the various design factors (most notably, L-DOPA and age), linear mixed models (LMM) were used to asses possible main effects and their interactions. Specifically, the model included fixed main effects of intervention (L-DOPA vs. Placebo), age group (OA vs. YA), ROI (EVC, RSC, HC), and session order (L-DOPA – Placebo vs. Placebo – L-DOPA), as well as their interaction. The random effects included a participant wise intercept and random slope of intervention. For models including data only from one ROI, the random slope of intervention had to be dropped to avoid singularity (same number of random effects as there are data points).

Additionally, we included several control factors in our models: A drug dosage relative to body weight and dosage/kg × intervention interaction tested for potential effects of body weight; and an effect of framewise displacement (FD) and an FD × intervention interaction were included in the model as a nuisance variable to capture possible effects of drug-related head motion.

*Influence of spatial angular difference on fMRI pattern similarity*

To test if neural representations of walking direction show the same circular similarity structure as directions in geometrical space, we analysed the structure of classifiers predictions as in Koch et al. (2020). If the similarity of two fMRI patterns of two different directions is associated with their angular distance in space, this should be reflected in the probability distributions over all possible directions. Specifically, we extracted the probability estimates of each of the six classes for each example of the testing set as calculated by the logistic regression classifier. These estimates were aligned with regard to relative angular difference from the target class ($-120^{\circ }$, $-60^{\circ }$, $0^{\circ }$, $60^{\circ }$, $120^{\circ }$, $180^{\circ }$) and then averaged over all examples, resulting in a single curve for each participant which we refer to as the *confusion function*. A simple Gaussian curve in the form of

(1) $$g\left(x\right)=\frac{1}{Z}e^{-\frac{1}{2}\tau x^{2}},$$

was used to determine tuning specificity, where x denotes the angular difference and τ the precision (the inverse of the variance, $\frac{1}{\sigma^{2}}$). Furthermore, Z normalizes the curve. This model captures an inverse relationship between the angular difference of two walking directions and the confusability of their associated neural patterns. Models were fitted separately within each participant and ROI.

The Gaussian model allowed us to assess age-differences in directional tuning specificity, which were captured by the precision parameter τ. A LMM identical to the one modelling classification accuracy described in the previous section was used to analyze differences in precision.

### 2.8 Behavioral analysis

Task performance during the feedback phase was measured by the distance error: the Euclidean distance between the true location of an object and the location the participant placed the respective object (measured in virtual meters; vm; 1vm = 62.5 Unreal units). Performance for each trial was given by the average distance error across all five presented objects within a trial (missing responses due to exceeding the time limit were excluded). Kolmogorov–Smirnov tests indicated that performance scores of YA were not normally distributed (D=0.169, p=0.010, D=0.064, p=0.881, for YA and OA, respectively; tested for performance on the last trial). To assure normality, the average distance errors in each trial were log-transformed (D=0.054, p=0.941, D=0.106, p=0.323 after transform for YA and OA, respectively). To assess the process of learning during the feedback phase of the task, we compared the difference between the first and last trial. Note that in light of non-linear learning curves we did not use a linear model across all trials on purpose. The difference between the two log-transformed measures was modeled using an LMM including the fixed effects of intervention (L-DOPA vs. Placebo), age group, and session order (L-DOPA – Placebo vs. Placebo – L-DOPA) as well as a random intercept of participant. Additionally, we compared performance after learning (last trial) with an identical LMM. Furthermore, group-level performance was compared to chance given by the average distance error assuming random responses for every object. To this end, we uniformly sampled $10^{5}$ possible locations within the circular arena. The task was then simulated 1000 times while each response of each participant was randomly drawn from the pool of possible locations. This yielded a distribution of 1000 group-means assuming random performance over a given trial and allowed a comparison of trial-specific group-means

Finally, we aimed to quantify the relationship between the specificity of direction signals and task performance to see if more specific direction signals allow better performance on the given task. To this end, we used previous LMMs of classification accuracy but added the regressor of performance in the last trial of the experiment. To assure normally distributed values the log-transformed performance variable was used. Furthermore, performance values were demeaned to eliminate a possible confound between age group and task performance. The FD-related nuisance regressors as well as the interaction between dosage per body weight and intervention were dropped from the model. To see if L-DOPA enhanced signal specificity in proportion to its enhancement of task performance the above model was adapted to predict the difference between sessions in classification accuracy (L-DOPA – Placebo). The increase in task performance was given by the session difference (L-DOPA – Placebo) of the log-transformed performance in the last trial of the task.

## 3 Results

### 3.1 Behavioral results

**Fig. 2:**
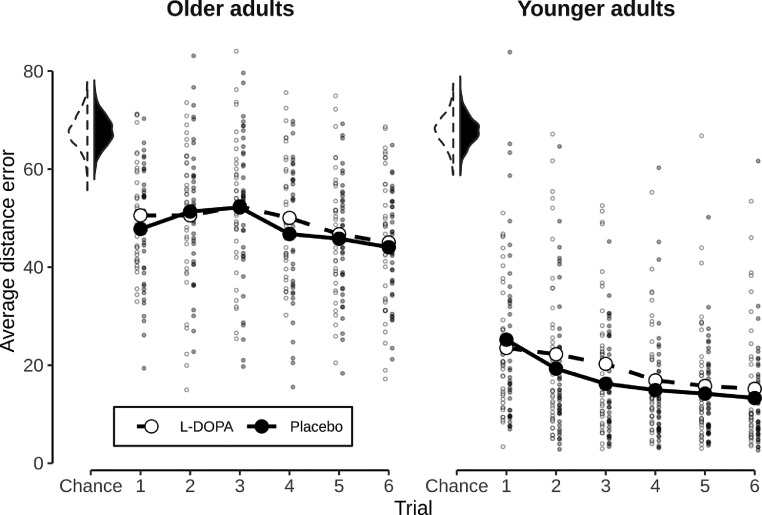
Behavioral results. Average error in object placement for all six trials for OA and YA. Error was measured as the Euclidean distance in vm between the true location of an object and the participants’ placement. Reduction in error shows better task performance. All values of the placebo session depicted in black, all values of the L-DOPA session depicted in white. Small dots indicate individual values of participants. Average over participants in each trial shown by the large dots. Shown on the upper left are session-specific distributions of 10^3^ average performance values in a trial assuming random placement of objects. Note that, in turn, only the trial averages (large dots) can be compared to this chance-distribution.

We first asked whether age group and intervention (L-DOPA vs. Placebo) affected participants’ object location memory, as expressed in distance errors on the last trial after learning. This this end, we ran a linear mixed model with fixed effects of interest for intervention and age group and a random effect of participant. This analysis showed a significant main effect of age group ($\chi^{2}\left(1\right)=167.010$, p>0.001; $\chi^{2}$ values reflect likelihood ratio tests, see Methods). Post-hoc tests showed that OA had higher distance errors compared to YA at the end of learning (t(80)=12.811, p<0.001). The model did not display any significant main effect of L-DOPA intervention ($\chi^{2}\left(1\right)=1.479$, p=0.224) or L-DOPA × age interaction. Results are displayed in Fig. 2.

We next investigated performance increases, i.e. log distance errors on the first minus the last trial, and again found only a main effect of age group ($\chi^{2}\left(1\right)=61.054$, p>0.001), but no main effect of L-DOPA or L-DOPA × age interaction. A control analysis showed that the nuisance variable session order had no main effect in either end-of-learning performance ($\chi^{2}\left(1\right)=0.1784$, p=0.673) or in performance changes ($\chi^{2}\left(1\right)=0.948$, p=0.330), and also revealed no session order × intervention effect in performance changes. Unexpectedly, we found a significant interaction of intervention × session order in end-of-learning performance ($\chi^{2}\left(1\right)=13.744$, p<0.001), reflecting a negative effect of L-DOPA if given in the first session (t(80)=3.368, p=0.002) while no effect was found if L-DOPA was given in the second session (t(80)=−1.693, p=0.180, Ŝidák corrected).

### 3.2 Influence of L-DOPA intervention on direction decodability

We used within-session cross-validation to investigate the decodability of walking direction (see Methods). A first analysis revealed that, averaged across sessions, decoding in our main areas of interest EVC (23.6%), RSC (18.4%) and HC (17.3%) was above chance baseline (16.6%, one-sided t-tests against chance, t(79)=11.783, p<0.001, t(79)=4.627, p<0.001, t(79)=2.011, p=0.047, respectively), while it was at chance in the entorhinal cortex (16.9%, p=0.257, all ps Bonferroni-Holm corrected for 4 ROIs). These results were largely confirmed by a permutation test, although the HC effect was borderline after correction (EVC: p<0.001, RSC: p<0.001, HC: p=0.058, Entorhinal Cortex: p=0.236, Bonferroni-Holm corrected).

Surprisingly, decoding in the left motor cortex was also above chance baseline, and significantly higher than in the HC (t(608)=−3.672, p=0.002) and entorhinal cortex (t(608)=−4.504, p<0.001). The high decoding score in the motor cortex was unexpected because participants used the same forward movement on the joystick to walk forward, regardless of the direction they traveled in. While we did not anticipate this effect, it indicates that this brain area cannot serve as a useful control ROI. The results of the motor cortex are depicted in greater detail in Inline Supplementary Figure S1, and will not be detailed further here. Note that correction for five instead of four ROIs does not qualitatively affect the results reported above.

**Fig. 3:**
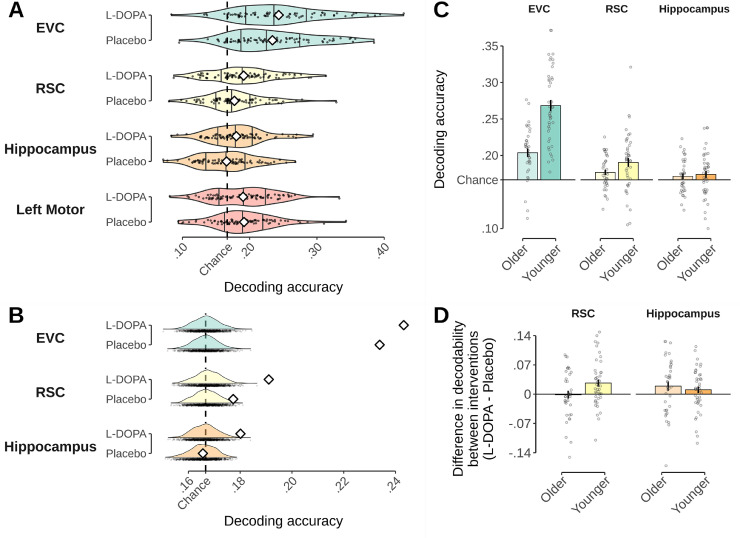
Effect of L-DOPA on decoding of neural walking direction signals. **A**: Intervention-specific decodability of walking direction within each ROI. Black dots show values of participants and violin plots depict intervention-specific distribution. Means are represented by white diamonds. Chance-level is shown by dashed line and based on the total number of classes (6 classes, 16.6% chance). **B**: Intervention-specific decodability of walking direction compared to chance baseline. Mean decodability in the sample shown as white diamonds. Distributions of 1000 sample means given shuffled labels during classifier training serve as chance baseline. Chance-level is shown by dashed line. **C**: Age group-specific decodability of walking direction. Dots show individual values of participants and bars show group averages. Error bars depict standard error of the mean. **D**: Influence of drug intervention on decodability (L-DOPA−Placebo) shown for the RSC and hippocampus and split by age groups. Values higher than zero indicate higher decoding accuracy in the L-DOPA condition. Bars reflect group means and error bars reflect SEM. Black dots show individual values of each participant.

Importantly, we next investigated whether classification accuracy was affected by dopamine, and indeed found a significant main effect of L-DOPA in a corresponding LMM ($\chi^{2}\left(1\right)=6.796$, p=0.009). This effect reflected that direction signals were generally stronger under L-DOPA than placebo (post hoc test: 19.5% vs. 18.6%, t(74)=2.556, p=0.013), in line with our main hypothesis (see Fig. 3A). Figure 3B shows permutation tests against chance baseline within the L-DOPA and placebo conditions. These permutation tests showed that decoding was above chance in both conditions in the EVC and RSC (EVC: p<0.001 & p<0.001, RSC: p<0.001 & p=0.036 for L-DOPA and placebo, respectively) while in the HC decoding was above chance only under L-DOPA (p=0.010), but not under placebo (p=0.884, all ps Bonferroni-Holm corrected). Control analyses testing the influence of nuisance regressors (FD, session order, dosage) in the LMM showed no main effects or interactions with the L-DOPA intervention (all ps >0.08). The LMM also indicated a number of other effects, in particular of age group ($\chi^{2}\left(1\right)=6.273$, p=0.012), ROI ($\chi^{2}\left(4\right)=271.674$, p<0.001), as well as an age group × ROI interaction ($\chi^{2}\left(4\right)=60.970$, p<0.001). But no L-DOPA × ROI or L-DOPA × age interactions were found (p=0.427 and p=0.506).

The main effect of ROI reflected that the classification achieved in EVC was significantly higher than decoding in the RSC (t(608)=10.837, p<0.001), HC (t(608)=13.108, p<0.001), left motor cortex (19.1%, t(608)=9.436, p<0.001), and entorhinal cortex (16.9%, t(608)=−13.940, p<0.001). In addition, decoding in the RSC significantly outperformed that in the entorhinal cortex (t(608)=−3.104, p=0.017).

Post-hoc comparisons of the age group main effect and the age group × ROI interaction showed that decoding was overall better in YA compared to OA, but this age difference was only significant in the EVC (t(359)=−7.424, p<0.001) but not in any other ROI (ps ≥0.833, Ŝidák corrected) as displayed in Fig. 3C. Note that the EVC also showed age differences in the size of the ROI, as reflected in significantly lower voxel numbers in OA compared to YA (p<0.001, 1583 voxels vs. 1361 voxels on average, respectively, p<0.001). However, repeating the decoding analysis in a subsample of participants matched for ROI size showed equally strong age differences in decoding, indicating that age differences in decoding found in the EVC are not explained by the larger EVC ROIs in YA (see Supplementary Materials Section 1 for details).

As noted above, no L-DOPA × ROI interaction was found. Our results therefore indicate that L-DOPA impacts the neural encoding of direction signals across a variety of brain regions. The following analyses therefore need to be seen as strictly exploratory. These exploratory follow-up analyses showed that the L-DOPA effect was strongest in the HC (t(603)=2.153, p=0.032), while post-hoc test in RSC and EVC revealed only marginal (t(603)=1.916, p=0.055), or non-significant effects (t(603)=1.447, p=0.148), respectively (all ps uncorrected). Neither the left motor cortex nor the entorhinal cortex did show any effects of L-DOPA (t(603)=−.211, p=0.833, t(603)=0.710, p=0.478, both uncorrected). To further explore trends in region-specific effects of L-DOPA, and interaction with age group therein, analyses were run separately for each ROI. These ROI-specific models reproduced the main effects of intervention within the HC ($\chi^{2}\left(1\right)=5.263$, p=0.022) and the RSC ($\chi^{2}\left(1\right)=4.868$, p=0.027). In addition, we found an intervention × age group interaction within the RSC ($\chi^{2}\left(1\right)=3.877$, p=0.049), but no such interaction in HC ($\chi^{2}\left(1\right)=1.518$, p=0.218, see Fig. 3D). Post-hoc comparisons showed that the effect in RSC was driven by higher decodability of walking direction in the L-DOPA compared to placebo session in young adults (t(75.6)=2.879, p=0.010), but not in OA (t(75.4)=−.161, p=0.984, Ŝidák corrected). Within the EVC, only a main effect of age group ($\chi^{2}\left(1\right)=16.350$, p<0.001), but no effect of L-DOPA intervention ($\chi^{2}\left(1\right)=2.038$, p=0.153) was found.

Control analyses found no impact of dosage per body weight on the intervention effect in any ROI ($\chi^{2}\left(2\right)<3.578$, p≥0.167, for the interaction). Investigating the movement related variable FD, we found no significant main effects of FD ($\chi^{2}\left(1\right)\leq 1.448$, p≥0.229) or an interaction between FD and intervention ($\chi^{2}\left(1\right)\leq 0.644$, p≥0.422) in HC or RSC. A significant main effect of FD was found in the EVC, however ($\chi^{2}\left(1\right)=4.935$, p=0.026). This reflected worse classification accuracy with higher movement during image acquisition (linear regression relating classification accuracy to FD: b=−.118, t(158)=−6.302, p<0.001). A final control analysis within the left motor cortex did neither identify a main effect of intervention ($\chi^{2}\left(1\right)=0.027$, p=0.869) nor any other main effects. Post-hoc tests confirmed that direction decodability in motor cortex under L-DOPA was not significantly different from decodability under placebo, regardless of session order (t(74.9)=−1.519, p=0.133, and t(74.1)=1.202, p=0.233, L-DOPA – Placebo and Placebo – L-DOPA, respectively).

### 3.3 Relations between task performance, L-DOPA and direction decoding

Following up on the above results, we asked whether neural direction encoding was related to task performance, and whether this relation was affected by L-DOPA. We therefore investigated the link between session-specific decoding accuracy and task performance (spatial distance error) on the last trial, in addition to age group and intervention. Because performance on the last trial was highly confounded with age group (see Fig. 2) performance values were demeaned within each age group to investigate effects unrelated to age-specific performance differences.

**Fig. 4:**
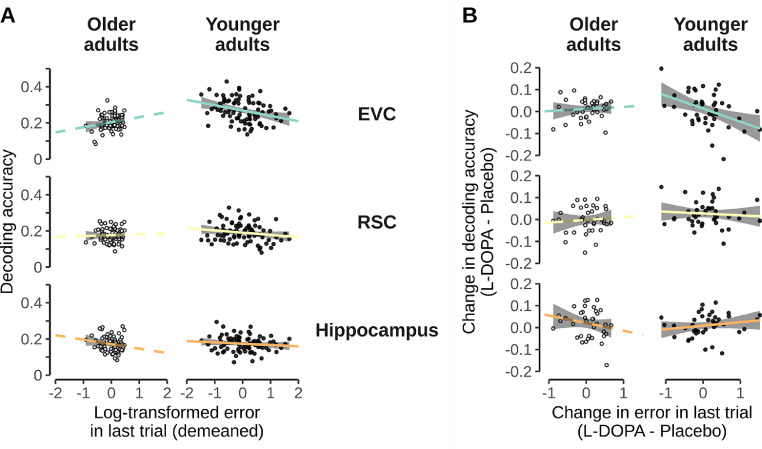
Relationship between decoding accuracy and behavioral performance. **A**: Relationship between decoding accuracy and log-transformed and demeaned distance errors. Shown for the EVC, RSC, and hippocampus separately for both age groups. Dots represent individual participants where OA are shown in white. Lines represent linear models of represented subset and are colored according to the ROI and shown in dashed for OA. **B**: Drug-related change-change relationship between decoding accuracy and behavioral performance. Axes show influence of L-DOPA administration by showing the difference in values between the L-DOPA session and placebo session. Depiction accordingly to A. Please note that in both, A and B, the slope lines were extended beyond the data points purely to aid visibility.

A model within the EVC revealed a significant main effect of distance error on the last trial on direction decoding ($\chi^{2}\left(1\right)=7.594$, p=0.006, b=0.040; see Fig. 4A), pointing towards better decoding accuracy with better task performance. The relation between task performance and EVC decoding also interacted with age group ($\chi^{2}\left(1\right)=3.921$, p=0.048), reflecting that the above mentioned relationship was present in YA (F(1,111.03)=11.912, p<0.001, b=0.033) and absent in OA (F(1,121.83)=0.066, p=0.798, b=0.006, both uncorrected). As expected the model of EVC decoding accuracy also displayed a main effect of age group ($\chi^{2}\left(1\right)=40.244$, p<0.001; see results for influence of L-DOPA on decoding accuracy). No effects related to task performance were found in the RSC or the HC (ps ≥0.053).

We next investigated change-change relations, asking whether L-DOPA-related changes in decoding were related to L-DOPA-related changes in task performance (see Fig. 4B). Linear regressions revealed that in YA L-DOPA-related changes in direction decoding in EVC were indeed positively related to changes in task performance (F(1,72)=6.730, p=0.011, b=−.053, uncorrected, negative slopes since performance increase means less errors). In OA, this was not the case (F(1,72)=0.049, p=826, b=0.006, uncorrected). Linear models within the RSC and HC did not show any significant effects in change-change relations. Hence, our results reveal that in EVC better direction decoding was related to better task performance. Moreover, the more L-DOPA improved direction decoding in EVC, the more participants improved on the task from the place to the L-DOPA session, in particular among younger adults.

A analysis of nuisance variables in EVC showed that there was no main effect of session order ($\chi^{2}\left(1\right)=0.009$, p=0.922), although a interaction between task performance and session order emerged ($\chi^{2}\left(1\right)=4.332$, p=0.037). A post-hoc test revealed a trend towards differing slopes depending if L-DOPA was given in the first or second session (t(132)=1.904, p=0.059) but separate tests within each session order did not display any significant relationships between performance and classification accuracy (F(1,143.83)=0.607, p=0.437, F(1,118.80)=3.164, p=0.078, for L-DOPA – Placebo and Placebo – L-DOPA, respectively).

### 3.4 Influence of L-DOPA intervention on tuning specificity

Finally, we investigated whether L-DOPA also affected tuning width, i.e. the how often neural signals encoding nearby directions where confused with each other.

**Fig. 5:**
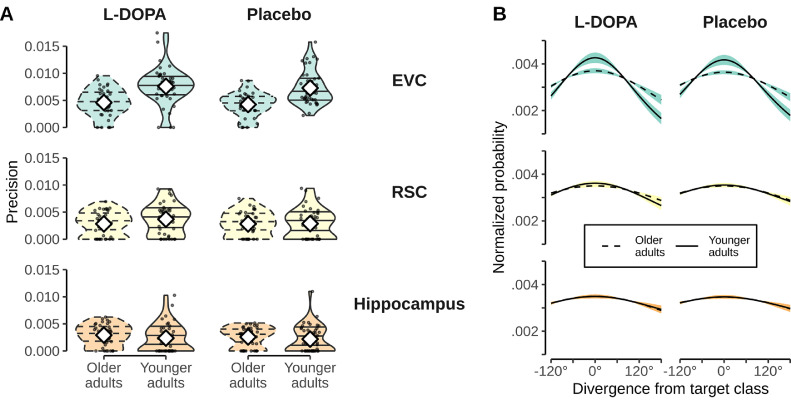
Effect of L-DOPA on tuning specificity. **A**: Precision of Gaussian curves fitted to individual confusion functions in both age groups. Shown separately for the L-DOPA and placebo intervention in the EVC, RSC, and Hippocampus. Black dots show values of individual participants. Intervention-specific distributions are shown by violin plots. White diamonds depict means. Plots of OA shown in dashed lines for easier distinction. **B**: Mean Gaussian tuning curves shown separately for age groups and intervention (L-DOPA vs. Placebo). ROI separation identical to that of panel A. OA are depicted with dashed lines. Shaded area represents SEM and is colored according to ROI. For each participant a Gaussian curve was fitted to the individual confusion function (given by the classifier). The shown mean Gaussian curves were obtained by averaging participants’ individual Gaussian curves.

Omnibus analyses across the main ROIs revealed no L-DOPA effect, a main effect of ROI ($\chi^{2}\left(2\right)=281.509$, p<0.001), and results otherwise consistent with those reported below. We therefore immediately report results of ROI-specific LMMs. A model of EVC tuning width found no main effect of intervention or intervention × age effect. We did find a significant main effect of age group ($\chi^{2}\left(1\right)=20.631$, p<0.001), reflecting lower precision of the fitted Gaussian curves in OA compared to YA (t(79.7)=−4.533, p<0.001). The same analyses in RSC and HC showed no significant main effects of intervention, age, or intervention × age interactions. The means of the fitted Gaussian curves in the L-DOPA condition are shown in Fig. 5B. Hence, L-DOPA did not have any effects on tuning functions in any of the investigated ROIs.

No nuisance effect of FD, session order, or FD × intervention interaction were found in any ROI-specific model ($\chi^{2}\left(1\right)\leq 0.857$, p≥0.355; $\chi^{2}\left(1\right)\leq 0.257$, p≥0.612, and $\chi^{2}\left(1\right)\leq 0.578$, p≥0.447, respectively). Additionally, intervention was not involved in any interaction with dosage per body weight ($\chi^{2}\left(2\right)\leq 4.412$, p≥0.110). Unexpectedly, however, we found a significant intervention × session order interaction in the EVC ($\chi^{2}\left(1\right)=10.713$, p<0.001; see Fig. 5A), suggesting that tuning precision was higher when L-DOPA was administered in the second session (t(74.0)=2.911, p<0.009) compared to when it was administered in the first session (t(75.2)=−1.607, p=0.212). No intervention × session order interaction was found in any other ROI.

## 4 Discussion

In this work we tested the impact of L-DOPA on neural representations of walking direction in younger and older adults, using a double-blind, cross-over intervention design. In addition to a classic decoding approach, we assessed direction specificity of neural signals, a proxy for tuning functions, using the relative structure of classifier probability estimates. Our results revealed that decodability of walking direction signals across all ROIs was enhanced following the administration of L-DOPA. Although no interaction between ROI and L-DOPA was found, post-hoc analyses hinted numerically at stronger effects in HC and RSC. Interestingly, however, task performance (spatial distance error) was related to EVC direction decoding in younger adults, and L-DOPA related changes in EVC decoding were related to changes in the same spatial memory measure. Moreover, these results showed that L-DOPA had comparable effects on HC walking direction signals in both age groups, but in the RSC these DA effects were present only in YA. An investigation of tuning specificity revealed no main effects of L-DOPA or L-DOPA × age group interactions.

Investigating age group differences, we found higher classification accuracy and precision of tuning functions in the EVC of YA compared to OA, a sign of neural dedifferentiation. No age effects on decoding in the HPC or RSC were found. These results confirm our previous finding that neural representations of walking direction can be found in EVC and RSC, and that strong age-related differentiation is present particularly in EVC Koch et al. (2020). We also showed that better EVC classification accuracy was related to better performance on task, suggesting an important functional role of this area in our task.

Importantly, our results also offer a number of novel insights. First, we show a causal influence of L-DOPA on how walking directions are encoded in the brain. No statistical evidence for ROI difference were found, but the pattern of results suggests that this effect was mainly driven by effects in the HC and the RSC. Hence, further investigations are needed in this regard. Both areas have been linked to directional and other spatial information (Burles, Slone, Iaria, 2017, Shine, Valdés-Herrera, Hegarty, Wolbers, 2016, Spiers, Barry, 2015), and have even been shown to be part of the same dorsal pathway involved in visuospatial processing (Kravitz et al., 2011). Additionally, both areas display dopaminergic innervation (Berger, Verney, Alvarez, Vigny, Helle, 1985, McNamara, Dupret, 2017), and previous reports have linked DA and spatial cognition more generally (El-Ghundi, Fletcher, Drago, Sibley, O’Dowd, George, 1999, Granado, Ortiz, Suárez, Martín, Ceña, Solís, Moratalla, 2008, Thurm, Schuck, Fauser, Doeller, Stankevich, Evens, Riedel, Storch, Lueken, Li, 2016). Notably, hippocampal decoding in the placebo session was at chance in both age groups, and only significant during the L-DOPA intervention. While the lack of decoding effects under placebo observed here might suggest that the human hippocampus under normal circumstances does not bear any information about traveling direction, this interpretation seems unlikely in light of the large literature suggesting otherwise (see Spiers and Barry, 2015, for a review). We therefore believe that the lack of effect may be due to issues of statistical power and noise in the data. In contrast to the placebo condition, the significant decoding results in the L-DOPA condition suggests that L-DOPA may have amplified existing directional signals in the hippocampus, rather than causing previously non-existent signals to appear de novo.

Second, the positive effects of DA on decoding are in line with computational models and empirical findings which suggest that DA affects neuronal gain (Cohen, Servan-Schreiber, 1992, Li, Rieckmann, 2014, Thurley, Senn, Lüscher, 2008). Accordingly, DA’s influence on neural gain could lead to a stronger separation between signal and noise, which made different stimuli more specific and easier to distinguish for the classifier. It should be noted, however, that we did not find any direct effects of L-DOPA on neural direction tuning specificity, which measures how similar neural patterns are to similar directions. Given the effects of DA on neural gain, we had hypothesized that this measure could be more sensitive to the effects of our intervention, but this was not the case. One possible explanation is that our design lacked the power to fully capture the neural tuning functions within just one session. Tentative analyses of EVC and RSC tuning specificity did show DA-related enhancement only in participants who received L-DOPA in the second session. We will discuss these session-specific effects further below. Third, our study was set up to ask whether the L-DOPA intervention might reduce age-related neural dedifferentiation. Virtual walking direction offered a promising window to answer these questions since it has previously been shown to be subject to age-related neural dedifferentiation (Koch et al., 2020) and the broader domain of spatial cognition has been shown to be highly age-sensitive (Lester, Moffat, Wiener, Barnes, Wolbers, 2017, Wolbers, Dudchenko, Wood, 2014). Age is also known to cause substantial loss of DA functioning (e.g. Bäckman et al., 2006), and we speculated that a lower baseline DA availability might magnify the effects of L-DOPA. Surprisingly, we did not find that the effects of L-DOPA were particularly pronounced in OA. Rather, the HC showed age-equivalent effects, and decoding in RSC was in fact enhanced only in YA.

Other than individual differences in baseline DA level, task demand may also affect the inverted-U function of DA modulation (Cools and D’Esposito, 2011). The spatial navigation task used in our study is quite demanding, such that YA though have higher baseline DA level could still benefit from the L-DOPA intervention, whereas in OA the task demand may still outweigh the benefit of L-DOPA intervention. While unexpected, these results could offer interesting insights into the complexity of how external DA medication might interact with neural differentiation and compensatory plasticity mechanisms that counteract age-related losses. One notable aspect in this regard is that we found no evidence of age-related dedifferentiation in HC or RSC, which speculatively could be a sign of compensatory mechanisms. It seems possible that DA interventions might only recover neural specificity in brain areas that are affected by age-related dedifferentiation. Contrary to this idea, we found no age-related L-DOPA effects in visual cortex, where dedifferentiation was observed – but this might be due to the relatively low D2 receptor density in this area (Lidow et al., 1989). Another possibility is that we did not observe age-specific effects of L-DOPA on neural direction encoding in RSC and HC for the same reasons we did not find age-related dedifferentiation in these regions. According to this idea, compensatory factors that have mitigated dedifferentiation also affected the effectiveness of external dopamine administration, for instance because of changed connectivity. Both ideas remain speculative and further studies are needed to fully understand how the effects of L-DOPA interventions on neural direction encoding interact with age and dedifferentiation.

Beyond these main implications, a number of interesting observation arose that warrant further investigation. Although we did not find any main effects of session order, we found some indications that session order could influence the effect of L-DOPA on neural signals that underlie spatial navigation. Age-differences in learning were stronger when L-DOPA was administered in the second compared to the first session. In addition, we found tuning specificity in EVC and RSC to be enhanced by L-DOPA only in participants who received the drug in the second session. Stronger effects when DA is administered in a second session have previously been reported in the context of spatial navigation (Thurm et al., 2016). The reason why session order effects could exist in this context are numerous. Garrett et al. (2015), for instance, highlight two possible explanations in the context of DA effects on neural signal variability. One is that previous training may increase the amount of baseline DA-release, based on findings in rodents (Owesson-White et al., 2008). A DA intervention could therefore lead to differing DA-availability depending on whether the participants had already been trained with the same or a similar task. A second possible explanation raised by Garrett et al. (2015) is that the environment is either learned in a state of higher or normal DA-availability. The state of the second sessions will consequently always be mismatched to the first session, leading to effects of drug administration given the respective session. Related to the first idea, we speculate that in our case general learning about the environment in a first placebo session could have established beneficial baseline for the effects of L-DOPA in the second session. Unfortunately, the present design is unfit to address such explanations and further evidence is warranted.

One open question is why the effect of L-DOPA on decoding in HC and RSC was not reflected in task performance, where no L-DOPA effect was found. In addition to generally small effects on neural representations, another explanation might be that task performance did not only depend on direction signals, but also relies on distance estimation and using distal and local cues, processes which themselves are affected by age (Schuck et al., 2015). The task might therefore have been too complex to provide a suitable behavioral measure. Interestingly, however, we did find some relationships between behavior and the specificity of directional information in visual cortex, indicating that neural markers might have different relations to performance in our task. This is shown by some of our results that also offer insights about age-related changes in the context of spatial navigation more generally. The results in the EVC showed that OA exhibit lower precision of directional tuning functions. This is a replication of findings reported in an earlier study using a similar analysis approach (Koch et al., 2020). During natural navigation and the perception of direction vision plays a major role as it allows stable directional signals (Goodridge, 1998) and corrects and prevents the accumulation of errors during path integration (Jeffery, 2007). A less precise visual signal in OA could therefore influence spatial signals downstream and contribute towards the pronounced difficulties OA have in spatial tasks. Interestingly, we also found a relationship between EVC direction decoding in YA and performance on task, suggesting better spatial memory performance if walking direction could be decoded with higher accuracy. While this concurs with previous reports of a link between (non-spatial) memory and signal specificity (Koen, Hauck, Rugg, 2019, Sommer, Fandakova, Grandy, Shing, Werkle-Bergner, Sander, 2019, St-Laurent, Abdi, Bondad, Buchsbaum, 2014), previous studies have mostly reported such links in older adults. Future work is required to further understand how age-related loss in specificity of visual signals might be involved in spatial cognition. That said, a simple propagation of less specific visual signals to the retrosplenial complex network seems unlikely, since there was no evidence for age-related dedifferentiation in the RSC or HC.

We would also like to point out a set of limitations that should be considered when interpreting the results of the presented work. While our results were statistically significant, and decoding performance compared to chance was broadly in line with previous studies (e.g. Koch, Li, Polk, Schuck, 2020, Shine, Valdés-Herrera, Tempelmann, Wolbers, 2019), the reported classification of direction signals remained numerically low in all ROIs. The substantial amount of wrong predictions of the classifiers even in the intervention session could indicate that effects of L-DOPA were small. We speculate that other factors influenced the BOLD signals that are unrelated to direction, including aspects related to vasculature, context or learning sensitivity of neural signals, and mixed selectivity of neural populations. In combination with a rather small number of training examples within each intervention session, this could explain the weak classifier performance. A second limitation is that the reported results come from a largely male sample, which questions whether our results generalize to women. Given the small sample size, the presented data also does not allow to draw conclusions regarding sex differences in spatial navigation, which have been reported in some (e.g. Andersen, Dahmani, Konishi, Bohbot, 2012, Spriggs, Kirk, Skelton, 2018; see Brake and Lacasse, 2018 for a review), but were absent in other studies (Bohbot, McKenzie, Konishi, Fouquet, Kurdi, Schachar, Boivin, Robaey, 2012, Levy, Astur, Frick, 2005, Rodgers, Sindone, Moffat, 2012). Another unexpected result was that we found substantial decoding performance in motor cortex. This is surprising given the fact that no one-to-one mapping between motor actions (joystick movement) and walking direction should exist (participants used the same forward movement on the joystick to walk forward, regardless of the direction they traveled in). One possible explanation is that joystick tilt was systematically related to travel direction, which would explain why this brain area carried direction information. Indeed, given that brain correlates of sensorimotor signals are often stronger and less noisy than correlates of abstracted quantities, the relative strength of decoding seems less surprising. In addition, this result may also speak to the fact that spatial navigation is supported by a wide network of brain areas, and hence a true control area might be less readily available. Of note, this decoding does also not seem to reflect an inflated chance baseline, since other areas showed no or significantly lower decoding, and no effect of L-DOPA in motor cortex was found (see SI Section 2). Future work is required to address these limitations and to in turn build a more concise framework in which our findings can be embedded.

In summary, we provide first causal insights into the role of dopamine in the encoding of spatial direction signals in the human brain. In addition, as suggested by exploratory data analysis, this enhancing effect of dopamine on the specificity of neural signals involved in navigation might mainly be present in the hippocampus and in the retrosplenial cortex, albeit there exclusively in younger adults. In combination with the replication of our own previous results (Koch et al., 2020), these findings offer insights into the neural processes underlying spatial navigation in the human brain, and how they are affected by age more generally.

## Data and Code availability

All code of the involved analyses will be published and made openly available at https://github.com/koch-means-cook/damson.

The data supporting the findings of this study will be shared in preprocessed or aggregated form that ensures full anonymity of subjects, in line with funding and ethical regulations. Given the drug intervention and double blind nature of this work, the precise constraints on data sharing are currently evaluated.

## Declaration of Competing Interest

The authors declare no conflicts of interest.

## Appendix A Supplementary materials

Supplementary Data S1 Supplementary Raw Research Data. This is open data under the CC BY license http://creativecommons.org/licenses/by/4.0/ Supplementary Data S1
