# Supplementary material for: L-DOPA enhances neural direction signals in younger and older adults
Source: Neuroimage. 2022 Dec 1;264:119670. doi: 10.1016/j.neuroimage.2022.119670 (PMC9771830; doi:10.1016/j.neuroimage.2022.119670)
Supplement: Supplementary Data S1 — Supplementary Raw Research Data. This is open data under the CC BY license http://creativecommons.org/licenses/by/4.0/ [file mmc1.pdf]

Supplementary Information

for

L-DOPA enhances neural direction signals in younger and older adults

Christoph Koch[1,2,*], Christian Baeuchl[3], Franka Glöckner[3], Philipp Riedel[4], Johannes Petzold[4], Michael Smolka[4], Shu-Chen Li[3,5], Nicolas W. Schuck[1,6,*]

[1] Max Planck Research Group NeuroCode, Max Planck Institute for Human Development, Berlin, Germany

[2] International Max Planck Research School on the Life Course, Max Planck Institute for Human Development, Berlin, Germany

[3] Faculty of Psychology, Chair of Lifespan Developmental Neuroscience, Technische Universität, Dresden, Germany

[4] Department of Psychiatry, Technische Universität Dresden, Dresden, Germany

[5] Centre for tactile internet with Human-in-the-Loop (CeTI), Technische Universität, Dresden, Germany

[6] Max Planck UCL Centre for Computational Psychiatry and Aging Research, Berlin, Germany, and London, United Kingdom

[*]Corresponding authors ([koch,schuck]@mpib-berlin.mpg.de)

1 Relationship between ROI size and classification accuracy

Each ROI was created from anatomical labels obtained from Mindboggle's FreeSurfer-based segmentation of each participant's individual T1-weighted images (Klein et al., 2017). Since the segmentation was conducted on individual images, the amount of voxels included in each ROI (i.e. size) varied between participants. Average size and variation of each ROI can be found in Table S1.

Table S1: Sample-average and standard deviation of number of voxels included in each ROI.

ROI	Mean number of voxels	SD
Entorhinal Cortex	174.09	27.86
EVC	1480.87	232.09
Hippocampus	323.64	28.68
RSC	198.55	32.14
Left Motor	555.45	71.59

As a control analysis we wanted to check if the number of voxels available for each subject within each ROI influenced classification accuracy. We set up five separate linear models, one for each ROI, relating classification accuracy to the number of voxels used to train and test the classifier (both variables z-scored). Decoding was significantly related to the number of voxels in EVC ($\beta = .342$, $R^2_{adj} = .106$, $F(1, 78) = 10.37$, $p = .002$, uncorrected) but no other ROI ($ps \geq .124$, uncorrected). Specifically, the model described a positive relationship so that higher classification accuracy was accompanied by a larger EVC ROI. The relationships are shown in Fig. S1.

Since the EVC was also the only ROI in which we reported age differences in classification accuracy, we investigated if this age difference in the EVC was related to differences in ROI size. Indeed, a two-sided t-test showed that older adults had smaller EVC ROIs compared to older adults (mean number of voxels OA: 1361.324, YA: 1583.744, $t(70.432) = -4.79$, $p < .001$). To test whether these age difference could explain age differences in classification, we created a subsample of 25 older and 25 younger participants with matched numbers of voxels within the EVC ROI. Specifically, we selected the 25 older adults with the highest voxel counts and then

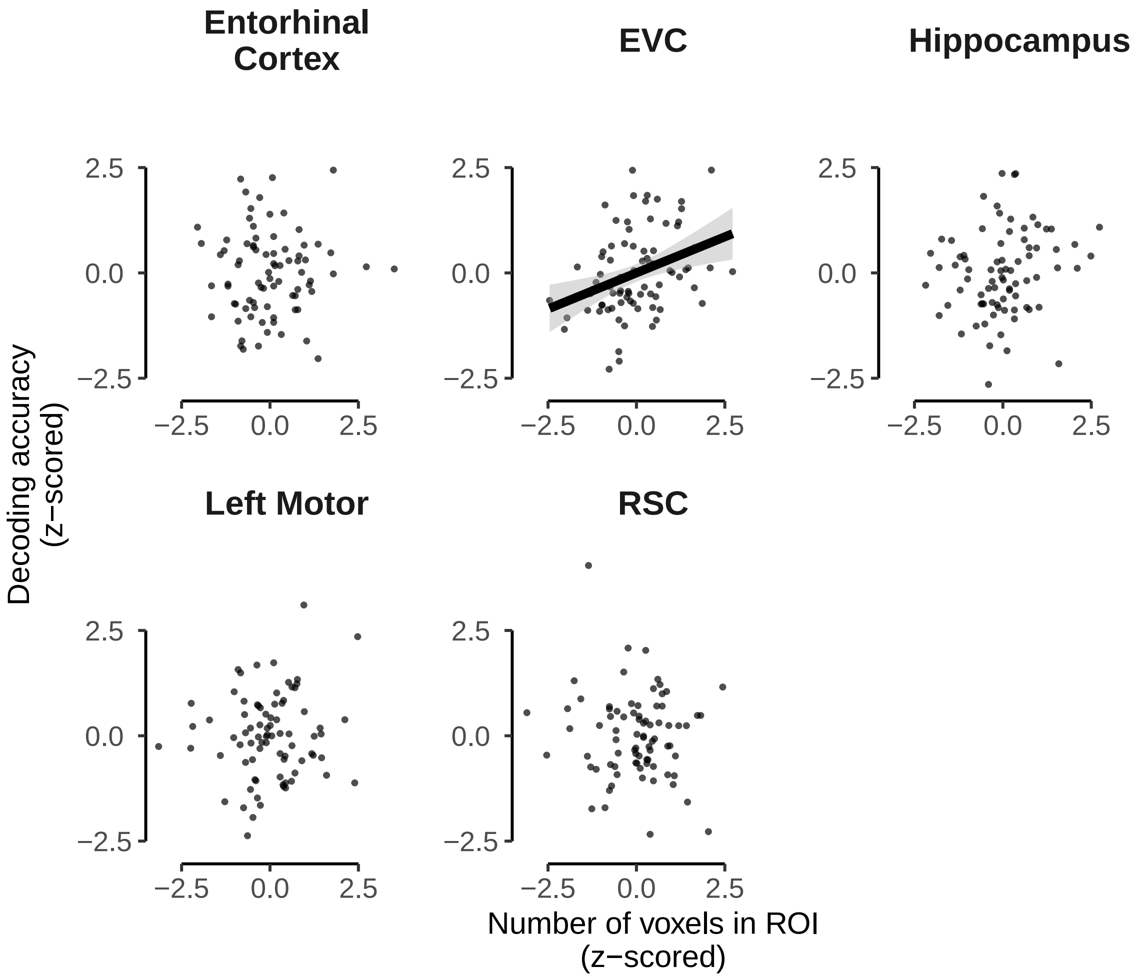

Figure S1: Linear relationship between decoding accuracy and number of voxels within each ROI (both variables z-scored within each ROI). Dots represent individual participants. Regression lines are only displayed for significant relationships.

picked 25 matched younger adults with the closest amount of voxels in the mask. This resulted in more comparable ROI sizes (older adults: 1463.56 voxels, vs. 1489.64 voxels in younger adults, $t(47.56) = -0.512$, $p = .612$). Importantly, a two-sided t-test still showed a significantly lower classification accuracy in older adults in the matched sample (diff = -.073, $t(45.25) = -5.62$, $p < .001$). We therefore conclude that the age differences in decoding found in the EVC are unlikely to be an artifact of larger EVC ROIs in younger adults.

2 Classification accuracy in left motor cortex

Permutation tests showed that average classification accuracy of direction across both sessions was significantly above chance in both age groups (OA: 18.5%, $p < .001$, YA: 19.6%, $p < .001$).

Further splitting up the data by age group and intervention shows that decoding is consistently above chance in all conditions (all $ps < .022$, uncorrected). Classifier performance for each intervention and age group is shown in figure S2. As reported in the main text (Results), no effects of intervention were found ($t(603) = -.211$, $p = .833$) and permutation tests confirmed these findings (test of true value against permutation distribution of 1000 differences between interventions given shuffled training labels, $p = .566$).

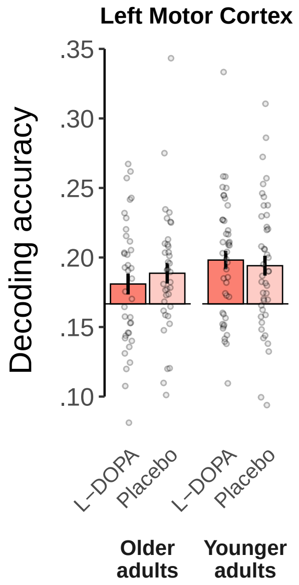

Figure S2: Classification accuracy of direction in the left motor cortex. Bars show average classification accuracy for each intervention and age group. Dots represent values of individual participants. Error bars show standard error of the mean.

3 Number of classifier examples between sessions and age groups

We first investigated systematic differences in the total number of classifier examples between age groups and sessions using a linear mixed effects model with a random intercept of participant. There was no significant effect of age group on the number of classifier examples ($\chi^2(1) = 1.335$, $p = .248$). The model showed a significant effect of session ($\chi^2(1) = 9.405$, $p = .002$), which described a lower number of events in the second session (on average 7.8 events difference) as revealed by post-hoc tests. This is likely to be caused by a training effect that the task might be solved more efficiently the second time resulting in less data due to a shorter navigation

time. More importantly, our analyses in the paper are based on the drug intervention, which was balanced across both sessions (counter-balanced intervention order: L-DOPA–Placebo or Placebo–L-DOPA). When running the same model with a fixed effect of intervention instead of session we found no difference in the number of events (mean number of events: 94.05 vs. 94.69 for Placebo and L-DOPA, respectively; $\chi^2(1) = .051$, $p = .822$). This model also did not display an effect of age group ($p = .248$). Furthermore, neither the model including the fixed effect of session, nor the model including the fixed effect of intervention showed a significant interaction with age group ($ps \geq .299$). When repeating the intervention analysis separately for each of the six directions only two of the six models showed marginal effects of age group. Because of the weak evidence for these effects and the high amount of comparisons made we did not interpret these findings as systematic differences in the number of classifier examples. Based on these findings, we are confident that differences in the number of classifier examples cannot explain our results.

References

Klein, A., Ghosh, S. S., Bao, F. S., Giard, J., Häme, Y., Stavsky, E., ... Keshavan, A. (2017). Mindboggling morphometry of human brains. *PLoS Computational Biology*, *13*(2), e1005350. doi: 10.1371/journal.pcbi.1005350
